# Effective Doses of Nalbuphine Combined With Propofol for Painless Gastroscopy in Adults: A Randomized Controlled Trial

**DOI:** 10.3389/fphar.2021.673550

**Published:** 2021-12-01

**Authors:** Shuangfeng Li, Ying Wang, Xiaojian Chen, Tingwan Huang, Na Li

**Affiliations:** ^1^ Department of Anesthesiology, Haikou Women and Children’s Hospital, Haikou Maternity and Child Health Hospital, Haikou, China; ^2^ Department of Anesthesiology, Hainan General Hospital, Haikou, China

**Keywords:** nalbuphine, propofol, gastroscopy, dose–effect relationship, ED50, ED95, effective dose

## Abstract

**Objective:** This prospective study evaluated the 50% effective dose (ED_50_) and 95% effective dose (ED_95_) of nalbuphine combined with propofol during painless gastroscopy.

**Methods:** Seventy-five patients who underwent painless gastroscopy were randomly divided into five groups (group N_0_, N_0.05_, N_0.1_, N_0.15,_ and N_0.2_), with doses of 0, 0.05, 0.1, 0.15, or 0.2 mg/kg nalbuphine in each group. Propofol was given to all groups as the sedative. The bispectral index (BIS) value, propofol dose, examination time, and awakening time were recorded. The number of patients with intolerance indexes (coughing, retching, swallowing, or limb movement) was recorded in each group. The ED_50_/ED_95_ of nalbuphine combined with propofol for gastroscopy were calculated.

**Results:** Compared with those of groups N_0_, N_0.05_, or N_0.1_, the propofol dose and awakening time were significantly reduced in group N_0.15_ or N_0.2_ (*p* < 0.05). The successful rate of painless gastroscopy in group N_0.15_ or N_0.2_ significantly increased compared to that of group N_0_ or N_0.05_ (*p* < 0.05). When combined with propofol, nalbuphine had an ED_50_ and ED_95_ for painless gastroscopy of 0.078 mg/kg (95% CI, 0.056–0.098 mg/kg) and 0.162 mg/kg (95% CI, 0.134–0.217 mg/kg), respectively.

**Conclusion:** The ED_50_/ED_95_ of nalbuphine combined with propofol are 0.078 and 0.162 mg/kg, respectively, for painless gastroscopy. Nalbuphine at 0.162 mg/kg combined with propofol is effective and safe for painless gastroscopy in adults.

## 1 Introduction

Gastroscopy is an efficient method for the diagnosis and treatment of digestive system diseases, including those of the throat, esophagus, stomach, and duodenum (Liu et al., 2015). Gastroscopy is a noninvasive operation, but gastroscopy without sedation/anesthesia often causes adverse reactions such as nausea, vomiting, and coughing (Meng et al., 2016). To provide patients with comfortable (i.e., painless) gastroscopy, propofol is considered the best sedation anesthetic according to many studies (Smith et al., 1994; Ellett, 2010; Olofsen et al., 2010; LaPierre et al., 2012; Zhang et al., 2014; Kılıc et al., 2016; Ma et al., 2016). Propofol has no analgesic effect; so, propofol combined with opioids can alleviate the cardiovascular response, reduce noxious stimuli, and reduce the dose of propofol (LaPierre et al., 2012; Anesthesiology Branch of Chinese Medical Association, Digestive Endoscopy Branch of Chinese Medical Association, 2014; Zhang et al., 2014; Kılıc et al., 2016). Too large a dose of opioid drugs could inhibit the patients’ respiratory and circulatory systems, and too small a dose of opioid drugs could not help patients to finish a gastroscope placement. Thus, exploration of the 50% effective dose (ED_50_) and 95% effective dose (ED_95_) of opioid drugs is useful to achieve a comfortable and safe gastroscopy for patients.

Nalbuphine is a κ-receptor agonist and partial μ-receptor antagonist. As a κ-receptor agonist, nalbuphine has a unique analgesic effect on visceral pain (Riviere, 2004), which is suitable for gastroscopy. As a partial antagonist of the μ-receptor, nalbuphine use may avoid a series of adverse reactions related to the activation of the μ-receptor (e.g., respiratory depression, addiction, euphoria, bradycardia, itching, immunosuppression, nausea and vomiting, intestinal peristalsis, and impaired bladder muscle function) (Misiołek et al., 2014). At the same time, the activity of nalbuphine on the δ-receptor is very weak; so, the drug does not produce irritability and anxiety (Misiołek et al., 2014). Low-dose nalbuphine has almost no effect on hemodynamics and respiration; so, it is very suitable for outpatient surgery (Lake et al., 1982). The application of nalbuphine in painless gastroscopy has a certain advantage over other opioids: nalbuphine acts quickly and safely (Liu, 2017). Finding the ED_50_/ED_95_ of nalbuphine combined with propofol will help patients have a safe, painless gastroscopy.

In this study, we evaluated the ED_50_/ED_95_ of nalbuphine combined with propofol during painless gastroscopy, and we determined the feasibility of nalbuphine combined with propofol for painless gastroscopy in adults.

## 2 Materials and Methods

This experiment has been approved by the Medical Ethics Committee of Hainan General Hospital (China, Reference No. 2018-103). Informed consent forms were signed by patients.

### 2.1 Patients

Overall, 75 patients who underwent painless gastroscopy in Hainan General Hospital from April 2020 to July 2020 were selected. The inclusion criteria were as follows: age 18–60 years, body mass index of 18–25 kg/m^2^, and American Society of Anesthesiologists classification I–II. The exclusion criteria included hepatitis and renal failure, habitual sedative or analgesic use, mental illness, and allergy to nalbuphine or propofol.

### 2.2 Clinical Protocol

The patients were randomly divided into five groups of nalbuphine according to dose, as follows: 0 mg/kg (group N_0_), 0.05 mg/kg (group N_0.05_), 0.1 mg/kg (group N_0.1_), 0.15 mg/kg (group N_0.15_), and 0.2 mg/kg (group N_0_._2_). All patients fasted from solids for 8 h and from liquids for 2 h. All patients took the left recumbent position in the operating room, with standard monitoring that included oxygen saturation (SpO_2_), noninvasive blood pressure, and electrocardiogram. All patients inhaled oxygen using the nasal catheter (3 L/min) for 5 min before anesthesia.

Nalbuphine (batch number: 1189503; Yichang Renfu Pharmaceutical Co., Ltd. Yichang, Hubei province, China) was injected intravenously at the onset; at 3 min after the onset, propofol (Batch number: RA170; AstraZeneca UK Limited, Macclesfield, Cheshire, SK10 2NA, United Kingdom) was administrated intravenously at a rate of 50–150 μg/kg/min until the patient lost consciousness, as reflected by a bispectral index (BIS) (BIS Complete Monitoring System, Covidien) value between 50 and 65. Then, the gastroscopy was performed. If the gastroscopy failed (defined as any coughing, swallowing, retching, or limb movement by the patient), the propofol dose was increased to facilitate completion of the examination. When the heart rate decreased to 50 beats per min, 0.2–0.5 mg atropine was injected. When the mean arterial pressure was less than 60 mmHg, 5–10 mg ephedrine was applied. When SpO_2_ decreased to 90%, assisted ventilation with oxygen via a facial mask was applied.

Observation indexes included the following: the BIS value at baseline (before the beginning of anesthesia) and at the beginning of gastroscopy, the effect-site concentration of propofol at the beginning of gastroscopy and at the end of gastroscopy, the propofol dose (the amount of propofol), the examination time (the time of gastroscopy), and the awakening time (the time between the end of the gastroscopy and when the patients to awake) were recorded. The number of patients with any coughing, swallowing, retching, or limb movement was recorded in each group. The number of patients who experienced a successful gastroscopy (defined as having none of the intolerance of coughing, swallowing, retching, or limb movement) was recorded in each group. The number of patients with apnea, postoperative pruritus, postoperative nausea/vomiting, or postoperative anxiety/irritability was recorded.

The effect index was the number of patients who experienced a successful gastroscopy, and the index was used to calculate the ED_50_/ED_95_ of nalbuphine combined with propofol. The patients who required increasing propofol doses were regarded as having a failed gastroscopy.

### 2.3 Statistical Analysis

IBM SPSS Statistics 23.0 (IBM Corporation, version 19; Armonk, NY, United States) was used for statistical analyses of data. The median effective dose (i.e., the ED_50_) and the ED_95_ as well as the 95% CIs of nalbuphine when combined with propofol were determined by binary regression (probit) (Gorges et al., 2017).

The required sample size was calculated using PASS 11.0 (Power Analysis and Sample Size; NCSS, LLC, Englewood, NJ, United States). The main indicator considered was the awakening time. The pilot study had 5 cases in each group. The mean ± standard deviation of awakening time in group N_0_ and group N_0.2_ were 726.13 ± 123.46 (s) and 245.00 ± 68.74 (s), respectively. A sample size of 15 in each group was required for a beta value of 0.10 and an alpha value of 0.05.

Normally distributed statistics were analyzed as the mean ± standard deviation and by one-way analysis of variance. Categorical variables were presented as proportions (%) and were compared using Fisher’s exact test or the chi-squared test. Trends in the nalbuphine doses were evaluated using the chi-squared test for trend. A *p* value of <0.05 was considered to be statistically significant.

## 3 Results

### 3.1 Included Patients Information

Overall, 77 patients were enrolled in the study, and 75 patients were assessed for eligibility. Two patients were excluded (one from group N_0_ and one from group N_0.15_) because gastric mucosa tissues were taken for observation by pathology and the duration of gastroscopy was more than 10 min. No significant demographic differences were noted among the five groups (*p* > 0.05). Results are shown in Table 1.

**TABLE 1:** Patient demographic characteristics.

	N_0_	N_0.05_	N_0.1_	N_0.15_	N_0.2_
ASA classification (I/II, n)	5/10	5/10	5/10	3/12	5/10
Sex (Male/female, *n*)	6/9	7/8	9/6	7/8	5/10
Age, years $\left(\bar{x}\pm s\right)$	42.46 ± 10.39	39.60 ± 10.20	38.53 ± 9.71	45.40 ± 8.26	40.20 ± 10.77
BMI, kg/m^2^ $\left(\bar{x}\pm s\right)$	23.14 ± 1.89	23.39 ± 1.71	23.03 ± 1.78	22.88 ± 2.16	22.38 ± 2.71

Note: ASA, American Society of Anesthesiologists; BMI, body mass index; $\bar{x}\pm s$ , mean ± standard deviation. One-way ANOVA was used to evaluate the differences in age and BMI. Sex and ASA classification were compared by the chi-squared test.

### 3.2 Intolerance Indexes: Coughing, Retching, Swallowing, and Limb Movement

Compared with group N_0_, group N_0.1_ had significantly decreased incidences of coughing (*p* < 0.05); groups N_0.15_ and N_0.2_ had significantly reduced incidences of coughing, retching, swallowing, and limb movement (*p* < 0.05). Compared with group N_0.05_, groups N_0.15_ and N_0.2_ had significantly reduced incidences of retching, swallowing, and limb movement (*p* < 0.05). Results are shown in Table 2.

**TABLE 2:** Comparison of intolerance indexes and successful gastroscopy incidences in five groups.

	N_0_	N_0.05_	N_0.1_	N_0.15_	N_0.2_
Coughing	7 (46.7%)	4 (26.7%)	0^#^	0^#^	0^#^
Retching	7 (46.7%)	7 (46.7%)	3 (20%)	0*	0*
Swallowing	13 (86.7%)	10 (66.7%)	6 (40%)	1 (6%)*	0*
Limb movement	13 (86.7%)	9 (60%)	6 (40%)	1 (6%)*	0*
Numbers of successful gastroscopy	1 (6%)	5 (33.3%)	9 (60%)^#^	14 (93.3%)*	15 (100%)*
Apnea	4 (26.7%)	2 (13.3%)	0	0	0
Postoperative pruritus	0	0	0	0	0
Postoperative nausea/vomiting	0	0	0	0	0
Postoperative anxiety/irritability	0	0	0	0	0

Chi-squared test and Fisher’s exact test were used to evaluate the differences among five groups. Compared with group N_0_, ^#^ *p* < 0.05; compared with group N_0_ or N_0.05_, *p < 0.05.

### 3.3 Adverse Effects

There was no significant difference in the incidence of apnea, postoperative pruritus, postoperative nausea/vomiting, or postoperative anxiety/irritability among the five groups (*p* > 0.05). Results are shown in Table 2.

### 3.4 Incidences of Successful Gastroscopy

Forty-four patients experienced successful gastroscopies (i.e., without any coughing, retching, swallowing, or limb movement). The incidences of successful gastroscopy in groups N_0.1_, N_0.15_, and N_0.2_ were significantly higher than those in group N_0_ (*p* < 0.01); compared with group N_0.05_, groups N_0.15_ and N_0.2_ had significantly increased incidences of successful gastroscopy (*p* < 0.01). No significant differences in success were noted among the other groups (*p* > 0.05). Results are shown in Table 2.

### 3.5 ED_50_ and ED_95_ of Nalbuphine Combined With Propofol

**FIGURE 1:**
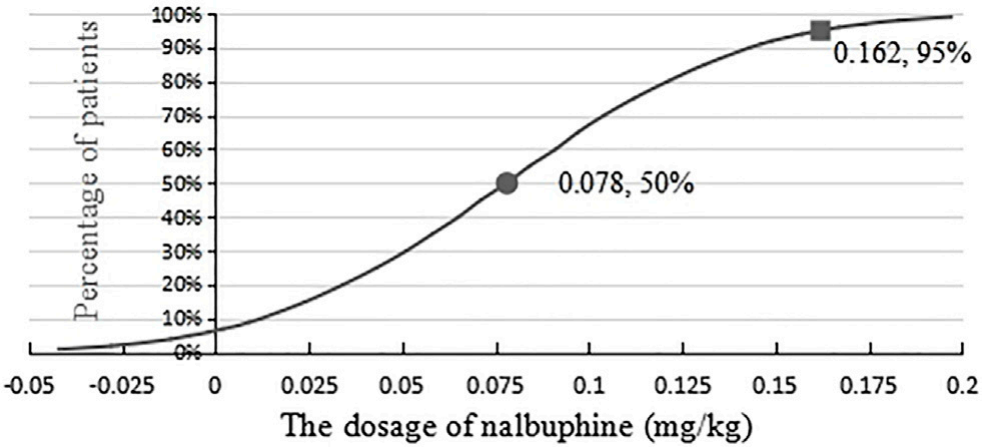
Effective dose of nalbuphine being combined with propofol anesthesia for gastroscopy in adults.

The statistics were analyzed by binary regression (probit). The ED_50_ of nalbuphine combined with propofol for painless gastroscopy was 0.078 mg/kg (95% CI, 0.056–0.098 mg/kg), and the ED_95_ of nalbuphine was 0.162 mg/kg (95% CI, 0.134–0.217 mg/kg) for the same procedure. Results are shown in Figure 1.

### 3.6 Comparison of the BIS Value, Effect-Site Concentration and Dose of Propofol, Examination Time, and Awakening Time

No statistically significant differences were observed in the BIS value at baseline or at the beginning of gastroscopy among the five groups (*p* > 0.05). After the nalbuphine dose was increased, the effect-site concentration of propofol, the propofol dose, and the awakening time decreased gradually in groups N_0_, N_0.1_, and N_0.15_ (*p* < 0.05), whereas no statistically significant differences were noted in these indexes between groups N_0.2_ and N_0.15_ (*p* > 0.05). The examination time for group N_0_ was significantly longer than that for any other group (*p* < 0.05), but no significant difference was noted among the other four groups (*p* > 0.05). All results are shown in Table 3.

**TABLE 3:** Comparison of the BIS value, propofol dose, examination time, and awakening time in five groups.

	N_0_	N_0.05_	N_0.1_	N_0.15_	N_0.2_
BIS value at baseline	97.40 ± 0.73	97.40 ± 0.73	97.40 ± 0.73	97.26 ± 0.59	97.00 ± 0.75
BIS value at the beginning of gastroscopy	58.80 ± 4.09	58.06 ± 3.05	58.26 ± 4.09	58.26 ± 4.51	60.13 ± 5.62
EC of propofol at the beginning of gastroscopy (ug/ml)	2.60 ± 0.37	2.42 ± 0.32	2.50 ± 0.45	2.34 ± 0.37	2.50 ± 0.41
EC of propofol at the end of gastroscopy (ug/ml)	4.18 ± 0.58	3.40 ± 0.78*	2.92 ± 0.73*^,#^	2.42 ± 0.41*^,#,△^	2.40 ± 0.39*^,#,△^
Propofol dose (1%, ml)	23.83 ± 3.22	19.66 ± 3.54*	16.32 ± 3.01*^,#^	12.29 ± 4.42*^,#,△^	11.64 ± 2.02*^,#,△^
Examination time (s)	225.33 ± 33.59	201.40 ± 28.92*	199.46 ± 30.95*	190.33 ± 33.96*	194.93 ± 25.73*
Awakening time (s)	740.13 ± 155.71	540.93 ± 205.37*	379.13 ± 198.69*^,#^	225.86 ± 105.52*^,#,△^	250.00 ± 64.45*^,#,△^

One-way ANOVA was used to evaluate the differences among five groups. Compared with group N_0_, *p < 0.05; compared with group N_0.05_, ^#^ *p* < 0.05; compared with group N_0.1_, ^△^ *p* < 0.05. BIS: bispectral index, EC: effect-site concentration.

## 4 Discussion

Although the surgery time for gastroscopy is short, it has a great impact on the physical and mental health of the patient. The anesthesia for gastroscopy requires a short time to the onset, adequate sedation and analgesia, quick recovery time after surgery, and low adverse effects. Gastroscopy often uses propofol combined with opioid analgesics. Nalbuphine, used in this study, is a κ-receptor agonist with a high concentration in the spinal cord. It produces mild respiratory depression, has hemodynamic stability, has a rapid onset, and is widely used in the clinic.

The basic requirement of anesthesia for gastroscopy is loss of consciousness and no body movement; so, this experiment set the BIS value at the ideal sedation level of 50–65 (Hao et al., 2014). According to the pharmacokinetic characteristics of nalbuphine, the time to the onset of effect is 2–3 min after intravenous injection (Cai et al., 2011); so, gastroscopy begins 2–3 min after nalbuphine injection to ensure that the analgesia of nalbuphine has taken effect.

The probit method (Cai et al., 2011) has been used to calculate the ED_50_ and ED_95_ of drugs. According to the requirements for the probit method, an arithmetic design of the 5 nalbuphine dose groups in this experiment was adopted. Dose selection was calculated according to results by Chen et al. (2018), who achieved an ED_50_ and an ED_95_ of nalbuphine during painless abortion of 0.068 mg/kg and 0.128 mg/kg, respectively. The probit method requires that the range of the selected dose in groups should cover the predicted value, and the result is more accurate when the arithmetic difference between groups is smaller. Therefore, intravenous doses of 0, 0.05, 0.1, 0.15, and 0.2 mg/kg nalbuphine were selected for this study.

In this study, six patients had apnea (4 in group N_0_ and 2 in group N_0.05_). If apnea lasts for more than 30 s, artificial respiration with a hand-controlled balloon was used to maintain blood oxygen saturation greater than 95% until spontaneous breathing was restored. The momentary apnea did not cause adverse effects for the patients. No patients had adverse cardiovascular reactions after receiving the anesthetic drugs; fluctuations of blood pressure and heart rate were within the physiological ranges, and cardiovascular drugs were not used.

In this study, as the dose of nalbuphine is increased, the total propofol dose is decreased; so, the awakening time of patients is shortened accordingly. It has shown that nalbuphine could be applied for patients safely in gastroscopy; no obvious respiratory depression, postoperative pruritus, postoperative nausea/vomiting, or postoperative anxiety/irritability occurred. Chen et al. (2018) compared nalbuphine with sufentanil—both in combination with propofol as anesthesia for abortion—and found that the awakening time in the nalbuphine group was significantly shorter than that in the sufentanil group. The incidence of dizziness (10%) in the nalbuphine group was significantly lower than that in the sufentanil group (33%) (Chen et al., 2018).

The incidences of coughing, retching, swallowing, and limb movement in patients decreased gradually with an increasing dose of nalbuphine. The reduction of these stress responses is conducive to successful gastroscopy. These results showed that the incidences of coughing, retching, swallowing, and limb movement in groups N_0.15_ and N_0.2_, with high-dose nalbuphine, were lower than those in groups N_0.05_ and N_0.1_, with low-dose nalbuphine. The number of successful gastroscopies in each group also increased with increasing nalbuphine dose: 1 case in group N_0_, 5 cases in group N_0.05_, 9 cases in group N_0.1_, 14 cases in N_0.15_, and 15 cases in group N_0.2_. Groups N_0.15_ and N_0.2_ had higher rates of successful gastroscopy than groups N_0.05_ and N_0.1_ had.

The probit regression method showed that the ED_50_ and ED_95_ of nalbuphine combined with propofol anesthesia for gastroscopy were 0.078 mg/kg (95% CI, 0.056–0.098 mg/kg) and 0.162 mg/kg (95% CI, 0.134–0.217 mg/kg), respectively. Therefore, 0.162 mg/kg of nalbuphine combined with propofol anesthesia is safe and effective for gastroscopy in adults, and it could provide the best conditions for gastroscopy, without any adverse reactions.

## 5 Conclusion

The ED_50_/ED_95_ of nalbuphine combined with propofol for patients with painless gastroscopy are 0.078 and 0.162 mg/kg, respectively. So, nalbuphine at 0.162 mg/kg combined with propofol is an effective and safe way for painless gastroscopy in adults.

This study had some limitations. First, no serum nalbuphine/propofol assays were used. Second, clinical examinations of outpatients were usually incomplete. Last, the hidden diseases and different sensitivities to drugs in individuals might have affected the results.

## Data Availability

The original contributions presented in the study are included in the article/supplementary material; further inquiries can be directed to the corresponding authors.

## References

[B1] Anesthesiology Branch of Chinese Medical Association, Digestive Endoscopy Branch of Chinese Medical Association (2014). Expert Consensus on Sedation/anesthesia of Gastroenterology in China. J. Clin. Anesthesiol. (China) 30 (9), 920–927. 10.3760/cma.j.issn.0254-1432.2014.08.001

[B2] Cai L. J. Zhang J. Peng W. X. Zhu R. H. Zhang Q. Z. (2011). Pharmacokinetics of Intravenous Nalbuphine in Healthy Volunteers. Chin. Pharm. J. (China). 46 (20), 1597–1600.

[B3] Chen L. Zhou Y. Cai Y. Bao N. Xu X. Shi B. (2018). The ED95 of Nalbuphine in Outpatient-Induced Abortion Compared to Equivalent Sufentanil. Basic Clin. Pharmacol. Toxicol. 123 (2), 202–206. 10.1111/bcpt.13022 29626849

[B4] Ellett M. L. (2010). A Literature Review of the Safety and Efficacy of Using Propofol for Sedation in Endoscopy. Gastroenterol. Nurs. 33 (2), 111–117. 10.1097/SGA.0b013e3181d601fb 20389224

[B5] Görges M. Zhou G. Brant R. Ansermino J. M. (2017). Sequential Allocation Trial Design in Anesthesia: an Introduction to Methods, Modeling, and Clinical Applications. Paediatr. Anaesth. 27, 240–247. 10.1111/pan.13088 28211193

[B6] Hao G. T. Zhou H. Y. Gao H. Z. Qu H. Y. Liang Y. G. Li Y. Y. (2014). Pharmacokinetics of Oxycodone Hydrochloride and Three of its Metabolites after Intravenous Administration in Chinese Patients with Pain. Pharmacol. Rep. 66 (1), 153–158. 10.1016/j.pharep.2013.08.012 24905321

[B7] Kılıc E. Demiriz B. Isıkay N. Yıldırım A. E. Can S. Basmacı C. (2016). Alfentanil versus Ketamine Combined with Propofol for Sedation during Upper Gastrointestinal System Endoscopy in Morbidly Obese Patients. Saudi. Med. J. 37 (11), 1191–1195. 10.15537/smj.2016.11.14557 27761556 PMC5303795

[B8] Lake C. L. Duckworth E. N. Difazio C. A. Durbin C. G. Magruder M. R. (1982). Cardiovascular Effects of Nalbuphine in Patients with Coronary or Valvular Heart Disease. Anesthesiology 57 (6), 498–503. 10.1097/00000542-198212000-00012 6983316

[B9] LaPierre C. D. Johnson K. B. Randall B. R. Egan T. D. (2012). A Simulation Study of Common Propofol and Propofol-Opioid Dosing Regimens for Upper Endoscopy: Implications on the Time Course of Recovery. Anesthesiology 117, 252–262. 10.1097/ALN.0b013e31825fb1b2 22728781

[B10] Liu J. Wang B. Hu W. Sun P. Li J. Duan H. (2015). Global and Local Panoramic Views for Gastroscopy: An Assisted Method of Gastroscopic Lesion Surveillance. IEEE. Trans. Biomed. Eng. 62, 2296–2307. 10.1109/TBME.2015.2424438 25910000

[B11] Liu Z. W. (2017). Anesthetic Effect and Safety on Propofol Combined with Nabufen Hydrochloride, Dezocine or Sufentanil Respectively for Painless Gastroscopy. J. Clin. Med. Lit. (China). 4 (29), 5708.

[B12] Ma J. Zhang P. Zhang Y. Chen Z. Xin W. Zhang D. (2016). Effect of Dezocine Combined with Propofol on Painless Gastroscopy in Patients with Suspect Gastric Carcinoma. J. Cancer Res. Ther. 12, C271–C273. 10.4103/0973-1482.200755 28230034

[B13] Meng Q. T. Cao C. Liu H. M. Xia Z. Y. Li W. Tang L. H. (2016). Safety and Efficacy of Etomidate and Propofol Anesthesia in Elderly Patients Undergoing Gastroscopy: A Double-Blind Randomized Clinical Study. Exp. Ther. Med. 12, 1515–1524. 10.3892/etm.2016.3475 27602075 PMC4998221

[B14] Misiołek H. Cettler M. Woroń J. Wordliczek J. Dobrogowski J. Mayzner-Zawadzka E. (2014). The 2014 Guidelines for post-operative Pain Management. Anaesthesiol. Intensive Ther. 46 (46), 221–244. 10.5603/AIT.2014.0041 25293474

[B15] Olofsen E. Boom M. Nieuwenhuijs D. Sarton E. Teppema L. Aarts L. (2010). Modeling the Non-steady State Respiratory Effects of Remifentanil in Awake and Propofol-Sedated Healthy Volunteers. Anesthesiology 112, 1382–1395. 10.1097/ALN.0b013e3181d69087 20461001

[B16] Rivière P. J. (2004). Peripheral Kappa-Opioid Agonists for Visceral Pain. Br. J. Pharmacol. 141 (8), 1331–1334. 10.1038/sj.bjp.0705763 15051626 PMC1574907

[B17] Smith I. White P. F. Nathanson M. Gouldson R. (1994). Propofol. An Update on its Clinical Use. Anesthesiology 81 (4), 1005–1043. 7943815

[B18] Zhang L. Bao Y. Shi D. (2014). Comparing the Pain of Propofol via Different Combinations of Fentanyl, Sufentanil or Remifentanil in Gastrointestinal Endoscopy. Acta Cir Bras 29 (10), 675–680. 10.1590/s0102-8650201400160008 25318000

